# Hepatic biomarkers and coprology as indicators of clinical bovine fasciolosis in Chad

**DOI:** 10.14202/vetworld.2021.1153-1157

**Published:** 2021-05-12

**Authors:** Ibrahim I. Elshahawy, Sherif Elshanat, Mayo Mahamat Arab, Mohamed Nayel, Akram Salama, Ahmed El-Sify, Mahmoud Aly

**Affiliations:** 1 Department of Animal Medicine, Faculty of Veterinary Medicine, Alexandria University, Egypt; 2 Department of Parasitology, Faculty of Veterinary Medicine, Alexandria University, Egypt; 3 Department of Biology, College of Exact and Applied Sciences, The University of N’Djamena, Chad; 4 Department of Animal Medicine and Infectious Diseases (Infectious disease), Faculty of Veterinary Medicine, University of Sadat City, Egypt; 5 Department of Animal Medicine and Infectious Diseases (Animal Medicine), Faculty of Veterinary Medicine, University of Sadat City, Egypt

**Keywords:** chad, coprology, fasciolosis, hepatic biomarkers

## Abstract

**Background and Aim:**

Fasciolosis is a cosmopolitan parasitic disease of food-producing animals and is typically caused by digenetic trematodes, *Fasciola gigantica* and *Fasciola hepatica*. It has a direct negative impact on the liver and consequently affects liver metabolism. It has indirect effects, including lowered milk production and effects on quality and general health conditions leading to extensive economic losses. This study aimed to focus on the link between clinical fasciolosis and some biochemical analysis of the hepatic profile of cattle in Chad.

**Materials and Methods:**

This study was initiated in response to emerging complaints from dairy cattle owners detecting a bitter milk cream and butter taste. Furthermore, those animals had shown poor health conditions by presenting with diarrhea. Preliminary surveillance for possible causes was performed, including fecal and serum biochemical analyses and clinical observation to diagnose the possible disease.

**Results:**

The results obtained, including the finding of parasite stages during the coprological examination, confirmed the role of fasciolosis. The independent sample t-test indicated highly significantly altered values of all biochemical liver indicators in the infected animals. All animals were treated with two doses of rafoxanide (3 mg/kg b.w.) S/C, at 21 days intervals, with vitamin supplements, mineral mixtures, and food additives. Surprisingly, the main complaint was restored after treatment. This is another evident clue of fasciolosis. To the best of our knowledge, this is the first recent study that diagnosed fasciolosis in Chad.

**Conclusion:**

This study emphasized the importance of fasciolosis, its negative impact on milk taste, and the necessity for veterinary advice regarding routine examination and prophylactic measures, especially before autumn, to minimize economic losses. However, regardless of the small sample size, this study could serve as a cornerstone for future studies on evaluating the accurate epidemiological status of fasciolosis in Chad. This study reported a close association between the alteration of liver enzymes and total protein levels in fasciolosis and the bitter milk cream taste, which could be used as a diagnostic tool for fasciolosis.

## Introduction

Fasciolosis, a parasitic disease affecting many food-producing animals worldwide, is caused by the digenetic trematode parasite *Fasciola gigantica* and *Fasciola hepatica* [1]. The infection is acquired following ingestion of encysted metacercariae on the grass during grazing, particularly near water sources. The main intermediate host, the freshwater snail of the family *Lymnaeidae*, is responsible for completing the life cycle of *Fasciola* spp. Globally, the food-producing animal industry is severely affected by fasciolosis, resulting in severe milk reduction and production in addition to its effect on fertility [2,3].

In some studies, a decrease in milk yield was observed due to *Fascioliasis* [4,5] alongside decreases in butterfat content. However, other studies have refuted these influences on milk [6,7]. Likewise, evidence of the impact of *F. hepatica* on fertility was controversial where some studies proved the reduction in animal fertility [4], whereas in other studies, there was no significant association between fertility parameters and *Fascioliasis* [5]. Furthermore, another cause of economic loss that is typically encountered is liver condemnation [8,9].

In addition to its direct effect on livestock, fasciolosis is also incriminated in increased susceptibility of coinfection with other pests [7,10]. In Chad, only a few studies have been conducted on fasciolosis. In the 1900s, only two studies were published on fasciolosis; one of them studied fasciolosis treatment [11], and the other was a collective Central African study that discovered the disease in Chad, around Lake Chad [12].

However, the first study on the association between *Fasciola gigantica* infection and geospatial distribution (climate, pasture conditions, and distribution of water bodies) of cattle, sheep, and goat was conducted in 2014. In this study, the author reported a higher risk of *F. gigantica* infection in animals grazing close to Lake Chad’s shore than that in animals far away from the lake [13]. Chad is a strong, appropriate environment for *Fasciola* spp. proliferation, especially with seasonal floods and increasing wetland areas. These climatic conditions create a suitable habitat for breeding of the intermediate host and parasite reproduction. Therefore, the rarity of publications on fasciolosis in Chad, coupled with complaints from some pastorals dealing with changes in milk taste, outlined this study’s aim. The activity of liver enzymes and other hepatic parameters was coupled in some cases with egg count for disease diagnosis [14]. Monitored elevation of aspartate aminotransferase (AST) and gamma-glutamyl transferase and elevated liver enzymes, hypergammaglobulinemia, and eosinophilia have been reported in the acute stage of *Fascioliasis* [15,16].

This study aimed to focus on the link between clinical fasciolosis and some biochemical analysis of the hepatic profile of cattle in Chad.

## Materials and Methods

### Ethical approval

This study was approved by the Institutional Animal Ethics Committee, Faculty of Veterinary Medicine, Alexandria University (Serial no: 12/10/2020/3/4/64).

### Study period and location

The study was conducted from May to July 2020. The animals under study were located in West Chad, the capital of ‘N’djamena. The samples were processed at Faculty of Veterinary Medicine, Alexandria University.

### Study overview and reason

The issue of bovine fasciolosis had come to our attention, especially after successful treatment of suspected cases of Fascioliasis resulting in low milk yield, bitter milk cream and butter, and diarrhea. Before we conducted the study, our suspicion was confirmed by finding eggs through fecal egg examination in some primary cases. Hence, on fecal egg examination, the administration of medications (rafoxanide (3 mg/kg b.w.) S/C, two doses at 21 days intervals) with vitamin supplements, mineral mixtures, and food additives. Then, we collected more samples to obtain a preliminary idea on fasciolosis status in the affected region and to study its effect on liver function.

### Study population and geography

The animals under study were located in West Chad, the capital of ‘N’djamena, where the Chari River runs through and most of Chad’s capital population lives around. First, the stimulus/need for this study arose from a few dairy cattle owners. The complaints included intermittent diarrhea, emaciation, and production of a small amount of milk with a bitter cream taste. Therefore, we randomly expanded the screening samples to precisely evaluate the case. Thus, 110 cattle were randomly collected at the beginning of autumn. The sample consisted of 58 dairy cows and 52 bulls; dairy cows were averagely aged 6-8 years, while bulls were averagely aged 3-4 years.

## Routine investigation

### Sample collection

All 110 Zebu cows were clinically examined to determine any clinical abnormalities. These include an examination of the mucus membrane, temperature, pulse, respiration, ruminal movement, animal coat, and udder for inflammatory signs. Furthermore, the case history was taken for each case; these were performed according to the described method [17]. Samples from each animal were collected under the same place and conditions. A variable number of samples were randomly collected monthly until 110 samples were collected over 3 months.

Blood samples were drawn from the jugular vein of each animal in plain tubes to use their serum for further chemical blood analysis. This gave us an idea of the vital functions inside the animal body, especially liver functions. All samples were collected before therapeutic interference. Fresh fecal samples were collected from each animal and kept in plastic bags. All bags were marked with a sample number, the owner’s name, time, and collection date. After that, all samples were moved to the laboratory for fecal examination within a maximum period of 48 h.

### Sample examination

All samples were grossly examined to detect the parasite growth stage, such as whole worms. Then, a part from each sample was subjected to conventional fecal examination. Each sample was examined using the concentration (sedimentation and flotation) technique, where the sedimentation method was performed using tap water, while a saturated salt solution with a specific gravity of 1.2 was used during floatation.

### Blood biochemical analysis

Sera samples were tested using a semi-auto biochemical analyzer (Sunostik Medical Technology Co., Ltd., Model SBA-733 Plus, Changchun, China). Most analyses investigated liver function, including tests to measure liver enzyme activity (alanine aminotransferase [ALT], AST, and alkaline phosphatase [ALP]). Other biomarkers for liver health, including bilirubin and total protein (TP), were measured, and a haptoglobin (Hp) measurement that acts as an inflammatory indicator was made. According to the manufacturer’s instructions, serum Hp was measured using a commercially available ELISA kit (Phase Hp kit, Tridelta Ltd., Ireland). Optical density was read at 450 nm using a computerized automated microplate ELISA reader (Bio TEC, ELX800G, USA). All measurements were made in duplicates.

### Statistical analysis

Statistical analyses were performed using an independent sample t-test to compare the infection diversity rate between the healthy and diseased groups. All data were represented as the mean±standard error of the mean. The statistical significance of the parameters was determined in the tests at p<0.001, represented by ***. Statistical analyses were performed using SPSS v.23 (IBM, Armonk, NY, USA), and graphs were created using Prism v.5 (GraphPad, La Jolla, CA, USA).

## Results

Coprological examination indicated that of 110 cases, 44 (26 female and 18 male cases) were *Fasciola* spp. positive (Table-1). However, of the same 110 cases, 50 (including 44 cases that were positive in microscopical examination) had elevated levels of the liver biomarkers tested, in case of inflammation. The study revealed 50 cases with abnormal ranges, indicating either ALT, AST, ALP, bilirubin, and Hp increase or TP level decreases. ALT, AST, ALP, and bilirubin were always higher than the upper reference limit and control ranges, except in six cases where bilirubin had a somewhat normal pattern. Without exceptions, in diseased animals, all biochemical liver markers had significantly altered values (p<0.001) compared to those in healthy animals (Table-2).

**Table-1:** General incidence rate (%) of *Fasciola* infection in examined cattle according to coprological examination.

Number of samples/sex	Number of positive	Number of negative	Percentage of positive cases
58 females	26	32	44.82
52 males	18	34	34.61
Total (110)	44 (all 44 cases showing elevated tested liver biomarkers)	66 (6 of them were showing elevated tested liver biomarkers except for bilirubin which was normal)	40

**Table-2:** The infection rate among healthy and diseased animals and their p-value and significance level, depending on analysis of biochemical liver markers.

Parameter	Healthy	Diseased	p-value	p-value summary
ALT	27.58±0.6200	78.89±6.463	<0.001	***
AST	101.9±1.363	199.5±14.52	<0.001	***
ALP	325.3±1.922	487.9±13.51	<0.001	***
Bilirubin	0.1206±0.006116	0.4411±0.02708	<0.001	***
TP	5.574±0.03795	4.789±0.09035	<0.001	***
Hp	0.04387±0.003065	0.1350±0.005899	<0.001	***

Significance level p<0.001 represented by ***. ALT=Alanine aminotransferase, AST=Aspartate aminotransferase, ALP=Alkaline phosphatase, TP=Total protein, Hp=Haptoglobin

**Figure-1:**
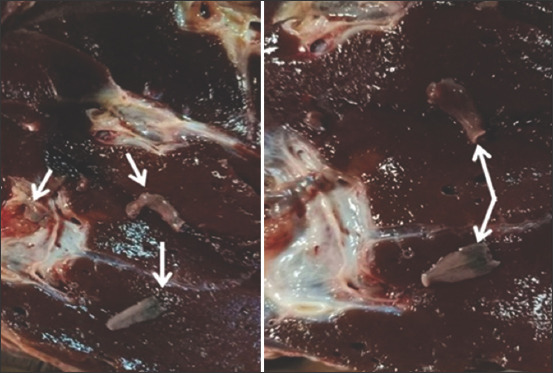
Live photo of whole worm of *Fasciola* spp., in bile duct in sacrificed animal.

Alongside coprological examination and biochemical analysis of blood, routine clinical examination was performed. Some degrees of manifestations were demonstrated, such as diarrhea, emaciation, and bitter milk cream and butter taste, which were the main complaints. The clinical signs were presented only in 38 cases (22 dairy cows and 16 males, two of these males were slaughtered because of severe ruminal stasis refractory to treatment). Adult *Fasciola* spp. were detected in their liver (Figure-1). However, most cases were normal. By linking the main complaint, bitter milk cream and butter taste, with abnormal biochemical analysis of liver-specific enzymes and coprological examination, we revealed that the main cause of the complaint was fasciolosis, which was also confirmed by lack/absence of prophylactic anthelmintic administration.

## Discussion

Fasciolosis is caused by two species, *F. gigantica* and *F. hepatica*. By contrast, other studies [18] found *F. gigantica* as the only cause of fasciolosis in Chadian cattle pastured along Lake Chad. Thus, fasciolosis is a major veterinary health problem. However, a proper control strategy of fasciolosis in Chadian cattle has yet to be considered. In this study, the general prevalence rate of fasciolosis in cattle grazing near Chari River was 40% compared to that in a study that recorded that 68% of investigated cattle were positive for fasciolosis around Lake Chad [13]. However, compared to our study, the aforementioned study involved large data. Both studies suggested that water bodies play a critical role in the transmission of fasciolosis. Coprology is sometimes not feasible [19]; therefore, a biochemical assay was used as a confirmatory diagnostic step. This revealed differences in serum liver biomarkers between normal and infected cattle.

The study revealed increased ALT, AST, and ALP, indicating that the liver is exposed. When these enzymes are realized in the blood, in case of hepatocyte injuries and necrosis, they serve as bioindicators of liver damage [20]. Furthermore, an increased total bilirubin value was observed in the infected animals because of hepatic function disturbance [21]. Bilirubin presence in milk has a powerful role as a lipophilic antioxidant [22], protecting membranes from lipid peroxidation [23,24]. Therefore, suggestively, an elevated bilirubin level in milk negatively affects the milk cream and butter taste. Furthermore, affinity to lipid linkage and higher level in the blood might reflect its content in milk. Moreover, a complete block or occlusion of the bile duct might play a critical role in its increase in blood and, consequently, in milk.

However, six cases indicated a normal bilirubin secretion with other abnormal liver biomarkers; it was assumed that the fluke did not completely occlude the bile duct, or the infection is still early. Furthermore, ALT and bilirubin disruption indicate severe liver damage. Moreover, the liver synthetic capability was evaluated, and TP was measured in the blood, with a low profile. TP is another blood parameter used to measure liver synthetic capability, because it is manufactured only in the liver [25]. Hp concentration was also evaluated; it was increased in all infected animals, where serum Hp indicates several inflammation types in cattle [26,27]. All animals, whether infected or uninfected, were treated with rafoxanide (3 mg/kg b.w.) S/C., and provided with some mineral mixtures and vitamins. The treatment of uninfected animals was considered to be a prophylactic measure, especially after realizing that no prophylactic programs had been extensively undertaken.

In this study, the clinical signs disappeared after flukicide administration, as reported by Charlier *et al*. [28]. This may be due to the enhancement of liver metabolism, regeneration of hepatocytes, or lowering of the parasite burden [5]. This study is insufficient to assess the epidemiological status of fasciolosis in Chad. However, it is a preliminary step to a further study where much data would be collected and statistically analyzed. Furthermore, *Fasciola* species should be identified, and pasture management on *Fasciola* infection transmission should be evaluated. From this study, regardless of the sample size, one significant issue that should be considered is that a prophylactic dose should be applied before the season when the parasite proliferates.

## Conclusion

The linkage between the activities of some liver parameters (as a laboratory observation), such as milk cream and butter bitterness (as a field complaint), and fasciolosis is considered to be a unique clinical aid, particularly in fasciolosis diagnosis. Climatic conditions in Chad create an appropriate breeding condition for the proliferation of *Fasciola* spp. Thus, it is strongly recommended that control measures, especially before the wet season, be considered, in addition to the importance of prophylaxis and pasture management.

## Authors’ Contributions

IIE and MA: Conceptualization. IIE, SE, and MMA: Methodology. IIE: Software. MN, AS, and AE: Validation. MA: Formal analysis. SE: Investigation. IIE, SE, and MMA: Resources. MN, AS, and AE: Data curation. IIE and SE: Writing – original draft preparation. MA: Writing – Review and editing. MN, AS, and AE: Visualization. MMA: Supervision. IIE and SE: Project administration. IIE, SE, and MA: Funding acquisition. All authors have read and approved the final manuscript.
